# Effect of pacifier and pacifier with dextrose in reducing pain during orogastric tube insertion in newborns: a randomized controlled trial*

**DOI:** 10.1038/s41372-024-01948-w

**Published:** 2024-03-29

**Authors:** Ayşenur Akkaya-Gül, Nurcan Özyazıcıoğlu

**Affiliations:** 1 Department of Child Health and Diseases Nursing, Health Sciences Institute, Bursa Uludağ University, Bursa, Turkey; 2 Department of First and Emergency Aid, Vocational School of Health Services, Fenerbahçe University, Istanbul, Turkey; 3 Department of Child Health and Diseases Nursing, Faculty of Health Sciences, Bursa Uludağ University, Bursa, Turkey

**Keywords:** Paediatrics, Pain management

## Abstract

**Objective:**

This study aimed to assess the efficacy of pacifier use, with and without 25% dextrose, in reducing pain during orogastric tube insertion in newborns.

**Study design:**

In a randomized controlled trial involving 60 newborns at a public hospital from April to December 2019, participants were divided into three groups: pacifier (*n* = 20), pacifier with 25% dextrose (*n* = 20), and control (*n* = 20). A pacifier, with and without dextrose, was used for the experimental groups, while the control group performed a routine procedure. Neonatal infant pain scale, crying duration, heart rate (HR), and oxygen saturation (SpO2) were evaluated.

**Results:**

Results indicated that the control group experienced significantly higher pain levels, elevated HRs, decreased SpO2, and prolonged crying. Conversely, the pacifier with 25% dextrose group showed a notable reduction in crying duration.

**Conclusion:**

A pacifier, with and without 25% dextrose, effectively reduces pain and improves physiological and behavioral parameters during orogastric tube insertion.

**Clinical trial number:**

NCT05462964

**Clinical trial registration:**

The protocol for this randomized controlled experimental trial is registered on ClinicalTrials.gov. The clinical trial registration number is https://clinicaltrials.gov; NCT05462964.

## Introduction

In the neonatal intensive care unit (NICU), infants undergo repetitive procedures for diagnosis and treatment [1–3] including feeding tube insertion as a common example [4, 5]. The feeding tubes are used in term and preterm newborns hospitalized in NICU due to immaturity of coordination of sucking, swallowing, and breathing or to respiratory, cardiovascular, gastrointestinal, or neurologic disease [6, 7]. These tubes enable nutrition, drug administration, decompression, and lavage during hospital stays [6, 8, 9]. Inserted via the nose (nasogastric) or mouth (orogastric), the tube carries insertion risks and stress for newborns [4, 10, 11], causing pain [8, 12–14].

Newborns depend on others to recognize, assess, and treat pain and discomfort [15, 16]. Nurses play a crucial role in pain assessment, interpreting behavioral, and physiological cues [17–19]. Pain assessment tools, such as the Neonatal Infant Pain Scale (NIPS), validate acute pain in term newborns [16, 20–22].

In the presence of measurable pain, it is necessary to reduce pain with effective pain management. Non-pharmacological interventions in neonates, like non-nutritive sucking and dextrose, enhance physiological stability and alleviate procedural acute pain [23–27]. Current approaches have shown that the use of the pacifier with sweet solutions (dextrose, sucrose) is more effective than its use alone [3, 26, 28, 29]. A systematic review suggests that the use of non-pharmacological methods, as well as in different painful procedures, feeding tubes (orogastric and nasogastric tubes) demonstrated that it was also effective in reducing acute pain during the insertion procedure [8].

Studies demonstrate the efficacy of non-nutritive sucking, dextrose, sucrose, breast milk, fetal position, and wrapping for reducing pain during orogastric and nasogastric tube insertion [12–14, 30–32]. Among these studies, it was observed that linguinal 25% dextrose [13], linguinal 24% sucrose [14], and breast milk combined with wrapping [30] reduced pain during the orogastric insertion procedure. The literature suggests that dextrose can be used as an alternative to sucrose solutions [29, 33, 34].

In the limited studies in the literature, the use of dextrose and pacifier alone had been discussed [12, 13, 32], but no study involving the combined use has been found in term neonatal. Unlike our study, only one study showed that using 30% sucrose combined with a pacifier in the orogastric tube (OGT) insertion procedure in premature newborns effectively reduced pain [12]. Existing studies predominantly focus on premature newborns [12, 14, 30, 31], with few addressing term newborns [13, 32]. This study contributes to the literature by expanding the subject to term newborns. In addition, the orogastric tube was advanced by sliding into the mouth of the newborn who was given a pacifier and a pacifier sweetened with 25% dextrose. It is hypothesized that this procedure will facilitate progress and reduce trauma by stimulating the swallowing reflex by means of a pacifier in newborns as well as in adults who are asked to swallow while the tube is being advanced.

Our study is thought to be important in providing new evidence to nurses in reducing pain due to orogastric tube insertion in term newborns. Our aim is to examine the effect of using pacifier and 25% dextrose pacifier on pain reduction during OGT insertion in newborns and to evaluate the effects on behavioral (crying time) and physiological parameters (heart rate (HR), oxygen saturation (SpO2)) in newborns.

## Methods

### Study design

**Fig. 1:**
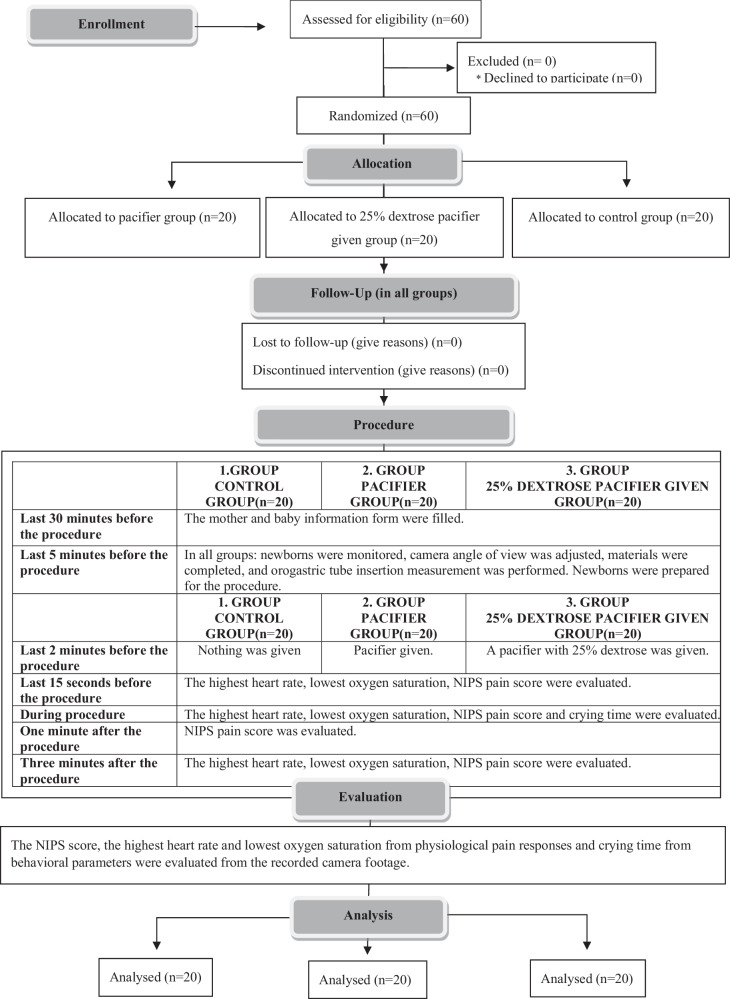
CONSORT 2010 flow diagram.

This study was conducted as a randomized controlled experimental study to examine the effect of using a pacifier and 25% dextrose pacifier on pain reduction during OGT insertion in newborns and to evaluate the effects on behavioral (crying time) and physiological parameters (HR, SpO2) in newborns. The CONSORT checklist was followed in reporting this study (Fig. 1).

### Research hypotheses

**Hypothesis 1:** The use of a pacifier alone and a pacifier induced with 25% dextrose in neonates has not an effect on reducing pain during and after orogastric tube insertion.

**Hypothesis 2:** The use of a pacifier alone and a pacifier induced with 25% dextrose in neonates has an effect on reducing pain during and after orogastric tube insertion.

**Hypothesis 3:** The use of a pacifier alone and a pacifier induced with 25% dextrose have not an effect on providing physiological stability during and after orogastric tube insertion in newborns.

**Hypothesis 4:** The use of a pacifier alone and a pacifier induced with 25% dextrose have an effect on providing physiological stability during and after orogastric tube insertion in newborns.

### Setting

This study was conducted in the NICU of Tuzla State Hospital in Istanbul, Turkey, between April and December 2019. The first researcher is a certified neonatal intensive care nurse for 6 years. Also a neonatal resuscitation program instructor. There are 11 incubators in the NICU and serve as first-stage. In Turkey, first-stage NICUs provide follow-up and treatment of babies with body weights over 2.500 g and in the risk group regarding newborn health. Newborns who are not at high risk are included. In Turkey, first-stage NICUs provide follow-up and treatment of babies with feeding difficulties, born with meconium, unable to maintain body temperature, in need of phototherapy, free oxygen support, respiratory distress is monitored with a monitor, needing non-invasive respiratory support whose hypoglycemia does not improve with enteral nutrition, and who require parenteral glucose support in terms of newborn health.

### Population and sample calculation

The population of this study consists of all term newborns hospitalized NICU in Tuzla State Hospital in Istanbul, Turkey. The sample included 60 newborns: 20 in the control group, 20 in one intervention group, and 20 in another. The study considered existing limited research on similar topics and used G*Power 3.1 for power analysis. Reference was taken from a crossover clinical trial [35] regarding pain scores during nasogastric tube insertion (oral sucrose 5.95 ± 2.35, routine procedures 9.93 ± 2.89). The number of samples was determined as a power range of 0.95 (1−β), an alpha level of 0.05 (α), and an effect size of 1.511(d), with each group of 13 newborns. To account for potential participant loss, each group had 20 newborns. No data loss occurred during the study.

### Randomization and blinding

Randomization was performed using the lottery method. Newborns were randomized during working hours every day of the week. A closed envelope was used for randomization. Incubator numbers of newborns with OGT insertion indication were placed in a sealed envelope. An independent employee drew lots. Afterward, they were assigned to the control group, the intervention group given the pacifier, and the intervention group given the 25% dextrose pacifier, respectively. The first researcher administering the intervention was not blinded to the intervention. Blinding was implemented only during the video evaluation stage. Support was received from the second researcher in the evaluations.

### Study inclusion and exclusion criteria

Term newborns at 38–42 weeks of gestation, whose feeding tubes were to be placed by mouth (orogastric tube), who did not receive painful stimuli until 30 min before the procedure, and whose parents volunteered to participate were included in the study. Newborns with any congenital anomaly on the face or oral cavity, newborns with 3rd- and 4th-degree intraventricular bleeding, who received muscle relaxants, analgesics, and sedation, as well as those with extreme prematurity, requiring invasive mechanical ventilation, or diagnosed with conditions such as cardiology, cardiovascular surgery, neurology, congenital anomalies, or severe perinatal asphyxia were excluded from the study.

### Data collection tools

Introductory information form of infant and mother, infant data evaluation survey (pain score, crying time, HR, and SpO2), and NIPS were used. A Nellcor Bedside console type pulse oximeter monitor was used to monitor the baby’s HR and SpO2, a camera focused on the baby’s face (iPhone 6S) that has the monitor in the frame with clear visibility, and a camera stabilizer (Xiaomi Bluetooth Tripod) that was used to keep it stable during the procedure were also set up for observations. Silicone pacifiers and 25% dextrose (obtained using 30% and 20% dextrose) were used for the intervention groups.

### Outcome measures and data collection

#### Primary outcome measure

The primary outcome measure was the pain score assessed by NIPS was developed by Lawrence et al. [21] and adapted into by Akdovan [36]. Akdovan, in her study, found that Cronbach’s α coefficient ranged from 0.83 to 0.86 [36]. NIPS comprises five behavioral (facial expression, crying, arm/leg movements, state of alertness) and one physiological (respiration) parameter. Scoring is 0–2 for crying, 0–1 for others, total score is 0–7. A high score indicates an increase in pain intensity. Evaluations were made from camera recordings pre-, during, and at the 1st and 3rd minutes of OGT insertion.

#### Secondary outcome measure

As secondary outcome measures, HR and SpO2 (within 15 s before OGT insertion, during the procedure, and 3 min after insertion) were considered in the physiological pain response assessment, and crying time (during the procedure) in the behavioral status assessment. The camera recording was watched, and recording was stopped at certain times, and evaluations were made.

### Intervention and control groups

Three groups were considered in this study. These are the control group (20), the first intervention group given a pacifier (20), and the second intervention group given a pacifier with 25% dextrose (20). A total of 60 newborns were included in the study. Two minutes before the procedure, newborns in the intervention groups were given a regular pacifier and a pacifier with 25% dextrose. Newborns in the control group were not given anything.

The pre-procedural stage was the same for all groups. The baby and mother introduction form, which was prepared using the literature information, was filled in before the procedure using the story form in the baby file. The newborns were placed under a radiant warmer in an open bed 5 min before the procedure. The procedure was performed in an incubator for newborns who could not be placed under a radiant heater. Before the procedures, the necessary materials were prepared. A number 6 or 8 (Fr) feeding tube was used for the procedure. OGT measurement was made, and the marking process was performed. A pulse oximetry (Nellcor Bedside) monitor probe was placed on the extremity of the newborn to monitor HR and SpO2. The newborns were prepared for the procedure by adjusting the camera focused on the baby’s face while having the pulse oximetry monitor within the field of view.

Camera recording was started 2 min before OGT insertion, and only a pacifier was given to the first intervention group and a pacifier with 25% dextrose to the second intervention group. In the OGT intervention groups, only the pacifier and the pacifier with 25% dextrose were placed in the mouth by sliding them from the side of the mouth. Then they were inserted into the esophagus and then into the stomach with the swallowing reflex. The first researcher performed orogastric tube insertion. Afterward, the OGT was checked for its placement. In the control group, the recording was started 2 min before the OGT insertion procedure, and routine OGT insertion was performed as in the other groups. After OGT insertion in the control group, a light touch was provided if necessary to ensure routine comfort of the newborn for ethical reasons. The recording was stopped after completing the evaluation period and ensuring the newborn comfort. However, audio and video recordings were continued to evaluate the crying time of the newborn who continued to cry during placement without time constraints.

Post-procedure evaluations were made by watching video recordings. The primary outcome measure of the study; the pain response score assessed by NIPS, the secondary outcome measure being the duration of crying, highest HR, and lowest SpO2.

### Analytical methods

Data normality was assessed using the Shapiro–Wilk test. Comparisons between groups were made by ANOVA and Kruskal–Wallis test. If the Kruskal–Wallis test result was significant, pairwise comparisons were made with the Dunn–Bonferroni test. Categorical data were compared with the Chi-square test. Changes from baseline and repeated measurements were made between groups by calculating percentage change values [percentage change = (last − before)/before]. A comparison of dependent groups was made with the Wilcoxon test. Descriptive statistics were mean ± SD for normal data and median (min–max) for non-normal data. Categorical data were presented as *n* (%). The significance level was *α* = 0.05. SPSS22 conducted analyses. An independent biostatistician performed the statistical analysis.

## Results

The study involved 60 newborns (20 in each group: control, pacifier only, pacifier with 25% dextrose). Demographic variables showed no significant intergroup differences pre-procedure (Table 1). Additionally, pre-procedural HR and SpO2 exhibited no significant variations between the three groups, establishing similarity (Table 1).

**Table 1:** Pre-procedure demographic characteristics and physiological parameters of newborns comparisons between groups.

	Groups (*n* = 60)			
Features	Pacifier group (*n* = 20)	25% dextrose pacifier given group (*n* = 20)	Control group (*n* = 20)	*p* value
Delivery method [*n* (%)]				
Vaginal	6 (30.0)	8 (40.0)	7 (35.0)	0.803^a^
Cesarean delivery	14 (70.0)	12 (60.0)	13 (65.0)	
Gender [*n* (%)]				
Female	8 (40.0)	7 (35.0)	9 (45.0)	0.812^a^
Male	12 (60.0)	13 (65.0)	11 (55.0)	
Apgar 1st minute				
(Median (min–max))	8 (5:8)	8 (3:9)	8 (7:8)	0.760^b^
Apgar 5th minutes				
(Median (min–max))	9 (7:9)	9 (5:9)	9 (8:9)	0.269^b^
Gestation age, wk				
(Median (min–max))	40 (38:41)	39.5 (38:42)	39 (38:41)	0.658^b^
Birth weight, g				
(Mean ± SD)	3301.75 ± 327.91	3423.00 ± 413.360	3493.75 ± 430.490	0.303^c^
Heart rate				
(Median (min–max))	142.5 (127:174)	145.5 (127:178)	135.5 (107:179)	0.162^b^
Oxygen saturation				
(Median (min–max))	97 (93:100)	98.5 (94:100)	96 (88:100)	0.096^b^

^a^Chi-square test was used for intergroup comparison of categorical data.

^b^Kruskal–Wallis test was used for the comparison between groups.

^c^Analysis of variance was used in the comparison of birth weight between groups.

### NIPS pain scores of the groups

The primary outcome measure of our study was the NIPS pain score. In our study, the NIPS pain score was found to be 0 in all groups before the procedure, since no painful intervention was performed in the newborns before the procedure. The groups are similar in this variable. NIPS pain scores of newborns during the procedure, 1 min after the procedure, and 3 min after the procedure were also compared between groups (see Table 2). Statistically significant differences emerged between control and intervention groups at all evaluation times. Paired tests revealed the control group as the source of these differences (Table 2).

**Table 2:** Intergroup comparisons of NIPS pain scores, heart rate, oxygen saturation, and crying time of newborns and pairwise comparisons of groups.

Evaluation time	Pacifier group (*n* = 20)	25% dextrose pacifier given group (*n* = 20)	Control group (*n* = 20)	*p* value^a^	Pairwise comparisons of groups *p* value^b^
During procedure NIPS (median (min–max))	4 (0:7)	1.5 (0:7)	6 (6:7)	<**0.001**	P–D = 0.185 **P**–**C** = **0.010** **D**–**C** = <**0.001**
One minute after the procedure NIPS (median (min–max))	0 (0:4)	0 (0:6)	5 (0:7)	<**0.001**	P–D = 1.000 **P**–**C** = <**0.001** **D**–**C** = <**0.001**
Three minutes after the procedure NIPS (median (min–max))	0 (0:7)	0 (0:7)	3 (0:7)	<**0.001**	P–D = 1.000 **P**–**C** = **0.003** **D**–**C** = <**0.001**
During procedure HR (%)	1.8 (−7:11)	1.8 (−4:9)	9.2 (−4:66)	**0.006**	D–P: 1.000 **D**–**C: 0.016** **P**–**C: 0.019**
Three minutes after the procedure HR (%)	1.7 (−16:29)	−2.1 (−15:12)	12.8 (−4:62)	<**0.001**	D–P: 0.826 **D**–**C: <0.001** **P**–**C: 0.013**
During procedure SpO2 (%)	−4.6 (−12:5)	−2 (−8:1)	−9.2 (−24: −1)	<**0.001**	D–P: 0.323 **D**–**C: <0.001** **P**–**C: 0.001**
Three minutes after the procedure SpO2 (%)	1.0 (−13:5)	0 (−7:4)	0 (−10:3)	0.089	
During procedure crying time	0 (0:10)	0 (0:5)	0 (0:60)	0.029	P–D: 0.374 P–C: 0.792 **D**–**C: 0.024**

P = pacifier group, D = 25% dextrose pacifier given group, C = control group.

^a^Kruskal–Wallis test, one of the non-parametric tests, was used for comparison between groups.

^b^Dunn–Bonferroni test was used for pairwise comparisons of the group.

Bold values signify statistical differences that are significant at the 0.05 level.

### Physiological parameters of the groups: heart rate and oxygen saturation

The study’s secondary outcomes included assessing the highest HR and lowest SpO2. These measures were recorded within 15 s before, during, and 3 min after the procedure. Pre-procedural HR and SpO2 showed no significant differences among the three groups (Table 1). Percentage changes in HR and SpO2 during and after the procedure were compared between groups, using pre-procedural values as a reference.

Statistically significant HR differences were observed between groups during and after the procedure compared to pre-procedure. Paired tests identified the control group as the source of this difference (Table 2). It was found that there was a statistically significant difference between the groups in SpO2 during the procedure compared to before the procedure. Paired tests identified the control group as the source of this difference (Table 2). There was no difference in SpO2 between the groups after the procedure compared to the pre-treatment (see Table 2).

### Behavioral parameters of the groups: crying time

Another secondary outcome measure was crying time during OGT insertion, compared among groups. It was found that there was a statistically significant difference between the groups. Paired test was performed to determine the group that created the difference. According to the pairwise comparisons of the groups: a statistically significant difference was found between the “control group” and “the pacifier group with 25% dextrose” (see Table 2).

## Discussion

Feeding tube (OGT/NGT) insertion is a commonly repeated procedure in both premature and term newborns, posing potential risks and discomfort [1–3]. Despite the necessity, OGT/NGT placement is known to induce pain and distress across all age groups, warranting effective pain management strategies [8, 37, 38]. Our study highlights the importance of minimizing pain during these procedures through non-pharmacological approaches.

This study showed that OGT insertion elicited a measurable pain response in term neonates [8]. Nurses have the responsibility to prevent or minimize pain during these practices. Using non-pharmacological methods (pacifier, dextrose, sucrose, breast milk, fetal position, and wrapping, etc.) during the feeding tube insertion process can make their insertion easier and faster, reduce adverse events, and increase patient and caregiver satisfaction [12–14, 30–32]. Our study aimed to examine the efficacy of using pacifiers and pacifiers sweetened with 25% dextrose for pain reduction during OGT insertion in newborns. Additionally, we sought to assess the impact on behavioral (crying time) and physiological parameters (HR, SpO2) in these neonates.

To the best of our knowledge, this randomized controlled experimental study is the first to examine the analgesic effect of using a pacifier and combined pacifier with 25% dextrose during orogastric tube insertion in term neonates. In the studies examined, the use of 25% dextrose and pacifier alone, which are non-pharmacological methods in reducing pain, were generally discussed, and no study involving the combined use was found. In a limited number of studies, 25% dextrose was used linguinally during OGT insertion [13] and 25% dextrose orally [32] during NGT insertion [13, 32]. There were a limited number of [13, 32] studies conducted on term newborns, with research focused especially on premature newborns [12, 14, 30, 31]. In this study, using a different technique, only the pacifier and the pacifier sweetened with 25% dextrose were in the mouth of the newborn, while the tube was advanced from the rim of the mouth and sent to the stomach with a swallowing reflex. In this method, it was observed that the pacifier stimulated the swallowing reflex during the OGT insertion process, resulting in easier progression.

The primary outcome measure was the NIPS pain score. Control group newborns had higher scores (Table 2), while intervention groups (pacifier and pacifier with 25% dextrose) experienced significantly less pain, confirming Hypothesis 2. While here was no statistical difference between the NIPS of the intervention groups, it is noteworthy that the use of 25% dextrose-sweetened pacifiers further reduced median NIPS scores, offering superior relief. Similarly, sucrose-sweetened pacifiers eased pain during NGT insertion in premature newborns [12]. The results show that the use of the pacifier with 25% dextrose and other sweet solutions (sucrose) is a more effective analgesic intervention than the use of the pacifier alone and aligns with other studies [3, 12, 26, 28, 29].

Furthermore, 25% dextrose is suggested as a sucrose alternative [29], proven pain relief and safe when used lingually [13] or orally [32] for feeding tube insertion. This result shows that as in our study, dextrose solution can be used safely as it activates in the mouth and does not require gastric and metabolic mediation. If dextrose was not available in our study, pacifier use alone was found to be a good alternative. Studies in the literature recommend pacifiers for repeated procedural pain, and our research supports this recommendation [23, 27, 39].

Newborns communicate pain non-verbally, employing behavioral (crying, movements) and physiological (HR, SpO2) cues [17–19]. Our study’s secondary outcomes encompassed crying time, peak HR, and lowest SpO2. Baseline HRs and SpO2 were statistically similar among newborns before the procedure (Table 1), aiding accurate intergroup comparison. In the intervention groups, HRs exhibited minimal change during and after the procedure compared to pre-procedure levels. Conversely, the control group experienced HR elevation during OGT insertion, persisting afterward (Table 2). Although newborns show physiological responses to pain, if unreliable pain persists, these responses may become erratic, and newborns may be at risk with poor outcomes. Consistent with our findings, a prior study noted increased HR among control group newborns during OGT insertion compared to those given 25% lingual dextrose [13].

Another physiological outcome was the lowest SpO2, which significantly declined during the procedure in the control group (Table 2). This simple intervention performed (pacifier and pacifier sweetened with 25% dextrose) had an essential role in physiological parameters in term newborns. This result confirms Hypothesis 4. These findings align with similar studies [13, 32]. In this study, oxygen saturations of all groups returned to pre-procedure levels after the procedure, and there was no difference between the groups [31]. This is a normal process, and no side effects (hypoxia, etc.) were encountered during the procedure.

Newborns express pain through crying, a biological response aiding adaptation to potentially harmful environments [37]. This study noted reduced crying time with a 25% dextrose-sweetened pacifier, while the pacifier alone didn’t show statistical significance (Table 2). Similar results were seen with 25% dextrose during NGT insertion [32]. These findings suggest dextrose’s calming effect warrants further discussion.

### Limitations

Our study, conducted in a primary care NICU, is subject to certain constraints. Limited personnel led to researcher performed orogastric tube placement without blinding. However, video recording was used for objective validation. Blinding was implemented only during the video evaluation stage. Monitoring research data via camera-recorded observations mitigated personal bias risk. Future research should involve larger samples, multiple centers, and potentially include preterm newborns for improved statistical precision and generalizability.

## Conclusion

This simple intervention (pacifier and 25% dextrose-sweetened pacifier) is clinically beneficial in reducing the pain burden associated with orogastric tube insertion and providing physiological and behavioral stability in term neonates. While the pacifier is in the mouth, it is a new method to advance the tube into the stomach by sliding it from the edge of the mouth. In this method, it was observed that the pacifier stimulated the swallowing reflex during the OGT insertion process, resulting in easier progression. As in our research, the sucking and swallowing reflex can be revealed by stimulating the baby’s palate with a pacifier, or the orogastric tube can be inserted while stimulating the baby’s palate with a finger. This method offers nurses an easy-to-use option to reduce infant pain and facilitate the advancement of the tube. Research on pain reduction during orogastric tube insertion should be increased in term neonatal.

### Recommendations for future studies

I have a few research ideas and suggestions for future consideration. Our research is a new method to advance the tube into the stomach by sliding it from the edge of the pacifier. Maybe it would be interesting to slide the orogastric tube through the center by pacifier puncture, include another group, and use breast milk instead of dextrose.

## Data Availability

The datasets generated during and/or analyzed during the study are available from the corresponding author upon reasonable request.
